# Highly selective hydrogenation of furfural to furfuryl alcohol over Pt nanoparticles supported on g-C_3_N_4_ nanosheets catalysts in water

**DOI:** 10.1038/srep28558

**Published:** 2016-06-22

**Authors:** Xiufang Chen, Ligang Zhang, Bo Zhang, Xingcui Guo, Xindong Mu

**Affiliations:** 1 Key Laboratory of Bio-based Materials, Qingdao Institute of Bioenergy and Bioprocess Technology, Chinese Academy of Sciences, Qingdao 266101, China

## Abstract

Graphitic carbon nitride nanosheets were investigated for developing effective Pt catalyst supports for selective hydrogenation of furfural to furfuryl alcohol in water. The nanosheets with an average thickness of about 3 nm were synthesized by a simple and green method through thermal oxidation etching of bulk g-C_3_N_4_ in air. Combined with the unique feature of nitrogen richness and locally conjugated structure, the g-C_3_N_4_ nanosheets with a high surface area of 142 m^2^ g^−1^ were demonstrated to be an excellent supports for loading small-size Pt nanoparticles. Superior furfural hydrogenation activity in water with complete conversion of furfural and high selectivity of furfuryl alcohol (>99%) was observed for g-C_3_N_4_ nanosheets supported Pt catalysts. The large specific surface area, uniform dispersion of Pt nanoparticles and the stronger furfural adsorption ability of nanosheets contributed to the considerable catalytic performance. The reusability tests showed that the novel Pt catalyst could maintain high activity and stability in the furfural hydrogenation reaction.

Furfuryl alcohol is an important chemical intermediate for the production of chemical products, such as vitamin C, lysine, plasticizer, dispersing agent, lubricant and resins^1^,^2^,^3^. Because of the importance of furfuryl alcohol in chemical industry and the manufacture of furfural from renewable resource, chemoselective hydrogenation of furfural to furfuryl alcohol has attracted great research interest. Due to the possibility of hydrogenolysis of C=O bonds, or decarboxylation, or hydrogenation of C=O bond or furan ring (Fig. 1), it is difficult to control the selectivity of the products. The catalytic hydrogenation of furfural to furfuryl alcohol is generally carried out either in the gas phase or in the liquid phase^3^,^4^,^5^,^6^. Compared with liquid-phase hydrogenation process, hydrogenation of furfural in gas phase would result in higher amount of byproducts and need higher energy consumption owing to the necessity of vaporizing furfural. Thus, it is attractive to explore an efficient catalytic system for furfural hydrogenation to furfuryl alcohol in liquid phase.

Catalytic hydrogenation of furfural to produce furfuryl alcohol in liquid phase had been widely investigated in the presence of Ni, Cu, Co, Pt and Pd based catalysts^5^,^6^,^7^,^8^,^9^,^10^. In the past, the Cu-Cr based catalysts were frequently used in industry, but Cr had great impact on the environmental pollution, limiting the further application^8^. Moreover, the liquid-phase hydrogenation reaction always processed in organic solvents, such as 2-propanol^5^,^10^, octane^6^, methanol^7^ and tetrahydrofuran^11^. The direct discharge of organic solvents would cause severe harm to both human bodies and environment. Undoubtedly, water is preferred from environmental points^12^. The use of water as solvent is more consistent with the goals of “Green Chemistry”. Thus, it is desirable to transform furfural to furfuryl alcohol at mild condition in aqueous solution and utilize less toxic components.

Noble metal nanoparticles have been demonstrated to be effective catalysts in the hydrogenation reaction^13^,^14^, which have been applied extensively in industry. Among the various noble metal nanoparticles, Pt-catalyzed hydrogenation reactions were widely used owing to the unique electrical and chemical properties of Pt catalysts^14^. Pt-based catalysts had also been employed in the furfural hydrogenation reaction^15^,^16^,^17^. For instance, Vaidya *et al*.^15^ used Pt/C as the catalyst for hydrogenation of furfural to furfuryl alcohol in a slurry reaction with 2-propanol containing 12.4% w/w water as solvent. However, a slow reaction rate and poor selectivity towards the desired furfuryl alcohol was obtained. The development of an active supported Pt catalyst with a high selectivity toward furfuryl alcohol, high stability and good recyclability remained a key challenge. Particle size is a key factor influencing the catalytic activity of Pt nanoparticles. Small size of Pt catalyst has advantages of high surface area and surface-to-volume ratio, which would be beneficial for improving catalytic efficiency. But Pt nanoparticles (<3 nm) would promote furfural decarboxylation to furan^18^. The Pt nanoparticles with smaller size also have high surface energy, and are easy to aggregate between interparticles, leading to poor stability and low utilization efficiency. Thus, dispersion of small-size Pt nanoparticles on suitable support materials is one of the best way of controlling and stabilization of Pt nanoparticles.

Among various supporting materials investigated, graphitic carbon nitride (g-C_3_N_4_) with graphite-like layered structure was the most promising catalyst supports for metal nanocatalysts, because of its advantages such as high chemical and thermal stability, unique electrical, and functional properties^19^,^20^,^21^,^22^. The nitrogen richness and locally conjugated structure endowed carbon nitride with anchoring small-size metal nanoparticles with high dispersion. The recent researches have shown that g-C_3_N_4_ supported Pd nanoparticles exhibited superior performance in several types of catalytic reduction, such as the hydrogenation of phenol^23^, nitrile^24^, quinolone^25^ and alkene^26^. For instance, Wang *et al*. firstly reported the hydrogenation of phenol to cyclohexanone over mesoporous g-C_3_N_4_ supported Pd catalysts with a high activity and selectivity^23^. Li *et al*. showed that g-C_3_N_4_ nanorods supported Pd nanoparticles could effectively reduce 4-nitrophenol with a high catalytic stability^27^. These results suggested that carbon nitride could be desirable supports for loading metal for hydrogenation catalysis. However, the catalytic performance of g-C_3_N_4_ based catalysts was usually limited for the low specific surface area. Some strategies have been attempted to overcome the disadvantage by controlling its nanometer-scale morphology or structure^26^,^27^. Recently, two-dimensional (2D) layered nanomaterials have attracted intensive attention for the high surface areas and distinct properties^28^. The intrinsic structural features made 2D layered nanomaterials highly desirable for potential application as catalytic supports for loading noble metal. Moreover, 2D layered nanomaterials usually had superior electron mobility, which would facilitate the electron transfer during the catalytic hydrogenation reactions. Compared with bulk g-C_3_N_4_, ultrafine g-C_3_N_4_ nanosheets had a large surface area, which facilitated the uniform dispersion of noble metal nanoparticles and good mass transfer effect. What is more, g-C_3_N_4_ possessed rich functional groups such as NH_2_, NH groups, and good hydrophilicity. The structural features of carbon nitride and 2D layered nanomaterials would make g-C_3_N_4_ nanosheets good dispersibility in water, which were highly advantageous in heterogeneous catalysis in water. However, the work on g-C_3_N_4_ based catalysts for the furfural hydrogenation reaction is almost scarce. Thus, it is attractive to fabricate metal nanoparticles supported on g-C_3_N_4_ nanosheets and study their application in the catalytic hydrogenation of furfural to furfuryl alcohol in water.

In this work, g-C_3_N_4_ nanosheets were synthesized from bulk g-C_3_N_4_ by a thermal exfoliation method, and the as-prepared nanomaterials were used as a basic support for loading Pt nanoparticles. The characterizations showed that Pt nanoparticles could be finely dispersed on the surface of g-C_3_N_4_ nanosheets. The application of this Pt supported catalyst in the furfural hydrogenation reaction demonstrated a superior catalytic activity and specific selectivity of the desired furfuryl alcohol. Particularly, the Pt supported catalyst could catalyze the hydrogenation of furfural efficiently in water and be reused for four times without obvious loss of activity and selectivity.

## Results and Discussion

### Characterizations of the g-C_3_N_4_ nanosheets supported Pt nanoparticles

A simple thermal exfoliation method was employed in the synthesis of g-C_3_N_4_ nanosheets^28^. Bulk g-C_3_N_4_ was first obtained by thermal polymerization of dicyandiamide at 550 °C. Then the bulk materials were delaminated to reduce the thickness to several nanometers by thermal oxidation etching process in air, thus yielding nanosheets. Finally, the as-prepared nanosheets were dispersed in water and adsorbed PtCl_6_^2−^ on the surface of g-C_3_N_4_ nanosheets, followed by reduction to Pt^0^ nanoparticles with NaBH_4_ as the reductant by an ultrasound-assisted method. The g-C_3_N_4_ nanosheets were donated as TECN. The obtained supported Pt samples were denoted as x%Pt@TECN, where x stood for the amount of Pt. The schematic diagram of the fabrication of Pt@TECN was illustrated in Fig. 2. As expected, the thermal exfoliation process caused the structure of carbon nitride materials to swell. It can be clearly seen from Fig. 3 that with the same weight of 100 mg, the nanosheets displayed much larger volume than the bulk materials. The structure of the as-prepared materials were investigated by XRD. Figure 4A exhibited that TECN had two main peaks at around 27.8° and 12.8° attributed to (002) and (100) peak of graphitic phase C_3_N_4_^21^, indicating that the typical graphite-like structure of C_3_N_4_-based materials was retained during the thermal exfoliation process. The results were supported by elemental analysis results, which showed that the atomic ratios of C to N in the bulk g-C_3_N_4_ and TECN were 0.66 and 0.68, respectively. A careful observation showed that the characteristic (002) peak derived from the interlayer stacking structure was shifted from 27.4° for the bulk materials to 27.8° for the nanosheets. The results suggested that the gallery distance between basic sheets was shortened after exfoliation, similar to the phenomenon reported before^28^. Figure 4B showed the XRD patterns of Pt@TECN with different Pt loadings. Apart from the characteristic peaks of carbon nitride, the two peaks at 40.0° and 46.2° were also observed in all Pt@TECN samples, assigned to the (111) and (200) peaks of metal Pt^0^ particles^29^,^30^. No other peaks were found in the Pt@TECN samples. Moreover, the Pt@TECN exhibited broad peaks of (111) and (200), and the intensity of Pt^0^ peaks enhanced with the increase of Pt contents, demonstrating the successful loading of small-size Pt nanoparticles on the surface of the carbon nitride support and no formation of impurities during the preparation of Pt catalysts. For the 5%Pt@TECN sample, the sizes of Pt particles were about 5.2 nm, calculated from the (111) peak according to the Scherrer formula. The results implied that g-C_3_N_4_ nanosheets could act as an excellent substrate to anchor Pt nanoparticles.

The surface elements and the chemical states of Pt species of 5%Pt@TECN were investigated by XPS. The XPS spectra in Figure S1A showed that C, N, O, Pt elements coexisted in the 5%Pt@TECN material, which was consistent with the XRD results. Figure S1B exhibited the high-resolution XPS spectra of C1s. The two main peaks with the binding energies of 284.6 and 288.2 eV were attributed to graphitic carbon and C-N-C coordination in TECN, respectively^22^. In the N1s spectrum (Figure S1C), three peaks at 398.7 eV, 399.9 eV and 401.3 eV could be deconvoluted, assigned to the C-N<C->C groups, tertiary nitrogen N-C3 groups and the amino functions carrying hydrogen (C-N-H), respectively^22^. The result of the Pt 4f was shown in Fig. 5. Four binding energies with two doublets can be separated. The stronger peaks at 71.1 and 74.4 eV for Pt 4f 7/2 and Pt 4f 5/2 were attributed to metallic Pt^0^. The other doublet at 72.4 and 76.0 eV could be assigned to Pt^2+^ states by surface layer formation of Pt-O or Pt-N bonds. The values were highly consistent with that of Pt^2+^ reported before^31^,^32^,^33^. The atom ratio of Pt^0^ calculated by XPS results was 71.3% in the total Pt atoms. The small amount of Pt^2+^ species might originated by surface layer formation of Pt-O or Pt-N bonds.

The textural properties of carbon nitride based catalysts were studied by nitrogen sorption. Figure 6 displayed the adsorption/desorption isotherm curves of bulk g-C_3_N_4_, TECN support and 5%Pt@TECN. As shown in Fig. 6, TECN showed a characteristic of type IV isotherm pattern, with a hysteresis loop of H3 type in the IUPAC classification. The result reflected the existence of mesoporous structure. As expected, TECN possessed a much larger surface area of 142 m^2^ g^−1^ than that of bulk material (10 m^2^ g^−1^). The pore-size distribution curve of TECN sample (Fig. 6, inset) showed that a mesoporous structure with the pore size in the range of 2–40 nm centered at around 3.5 nm was formed after thermal oxidation etching process, which was possibly formed by random-stacking of the nanosheets. These data illustrated that thermal oxidation etching could effectively increase specific surface area by exfoliating and thus form nanostructures. After deposition of Pt nanoparticles, the Pt@TECN still had a large surface area of 137 m^2^ g^−1^. The textural properties of the Pt@TECN was basically consistent with that of TECN support, suggesting that the Pt nanoparticles did not block the pore distribution of nanosheets. The Pt content in the 5%Pt@TECN determined by AAS analysis was about 4.5 wt%, close to the theoretical values.

The AFM was employed to analyze the morphology and the thickness of the nanosheets. The typical AFM image of TECN displayed flat and lamellar structures with a relative uniform thickness of about 3 nm (Fig. 7, right). For comparison, the bulk g-C_3_N_4_ showed the thickness of 5–25 nm with the stacked structures (Fig. 7, left). The results demonstrated that the layered material of g-C_3_N_4_ was successfully exfoliated into lamellar nanosheets with the average thickness of ~3 nm after thermal oxidation etching process. It is reported that the layers of carbon nitride were connected each other by weak van der Waals forces in the form of hydrogen-bond^28^. The interactions was not stable enough against thermal oxidation process in air, thus the layers would be exfoliated gradually away from the parent layered material to reduce their thickness to several nanometers, finally yielding the lamellar nanoscale structure. Moreover, g-C_3_N_4_ possessed rich functional groups such as NH_2_, NH groups on the surface, and good hydrophilicity. Nanosheet structure would make the carbon nitride better dispersibility in water. In order to illustrate the dispersion properties of carbon nitride in water, TECN and bulk g-C_3_N_4_ sample was suspended in water by sonication for 10 min and then put it aside at room temperature. As shown in Fig. 8, it can be observed that the nanosheets can be dispersed well in water by forming stable “milky” suspensions, even after storage for 6 h. In contrast, the bulk materials were more likely to precipitate at the same condition.

The morphology was further investigated by SEM shown in Fig. 9. The representative SEM image of the nanosheets (Fig. 9B,C) displayed soft and fluffy morphology with a size of several micrometers, different from that of bulk material consisted of solid agglomerates (Fig. 9A). The carbon nitride materials with the nanosheet architecture were superior carriers for small-size noble metal loading. For the Pt supported on TECN sample, the Pt could be well dispersed on the surface of nanosheets, which was confirmed by the elemental mapping images (Fig. 9F). The microstructure of bulk g-C_3_N_4_, TECN and 5%Pt@TECN nanomaterials was also studied by TEM. As shown in Fig. 10B, the morphology of TECN tended to flake rather than to aggregate together with large sheets. The fluffy and lamellar structures of TECN support were in good agreement with the AFM and SEM. TEM image of 5% Pt@TECN sample (Fig. 10C) also exhibited uniform distribution of Pt nanoparticles on the TECN support with an average particle size of 4.25 nm (Fig. 10D), similar to the calculated results from XRD (5.2 nm). It was believed that such nanosheet structures had the merits of offering a large surface area, providing more active sites and favoring the rapid diffusion of reactant and product during catalytic process. The reason for the well dispersion of Pt nanoparticles on the surfaces of TECN is partly attributed to the large specific surface area. The nanosheet structures would also be beneficial to enhance the adsorption ability of the substrates on the surface of the catalyst, which was further supported by furfural adsorption measurement. The adsorption capacities of both bulk g-C_3_N_4_ and nanosheets with furfural were measured at 25 °C and the results were shown in Fig. 11. The furfural adsorbed over TECN could reach 51 mg/g after adsorption for 6 h, which was higher than that of the bulk material (42 mg/g).

### Hydrogenation of furfural with Pt-based catalysts

**Figure 1:**
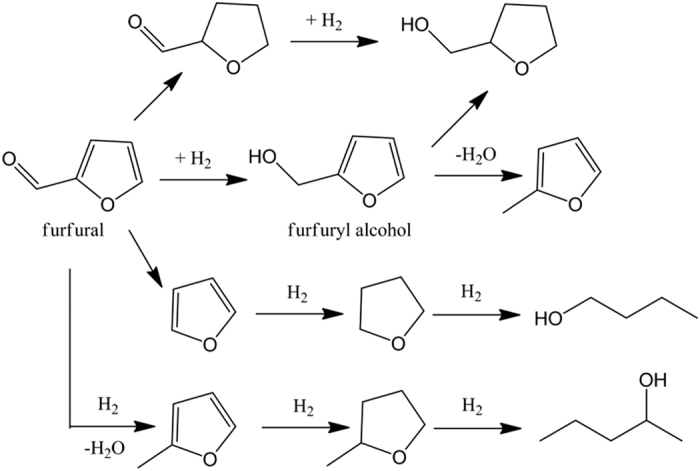
The schematic pathway for the hydrogenation of furfural.

**Figure 2:**
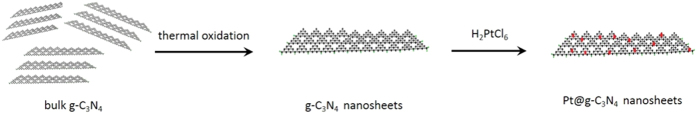
The schematic illustration of the fabrication of Pt@TECN.

**Figure 3:**
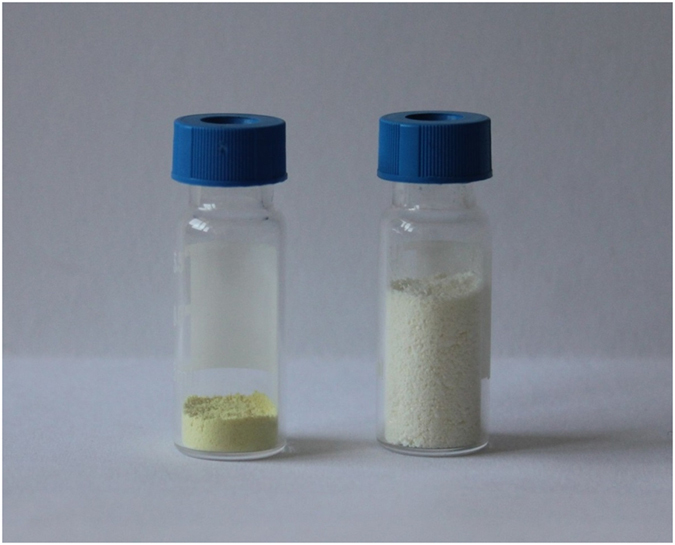
The volume comparison of 100 mg powder of bulk g-C_3_N_4_ (left) and TECN (right).

**Figure 4:**
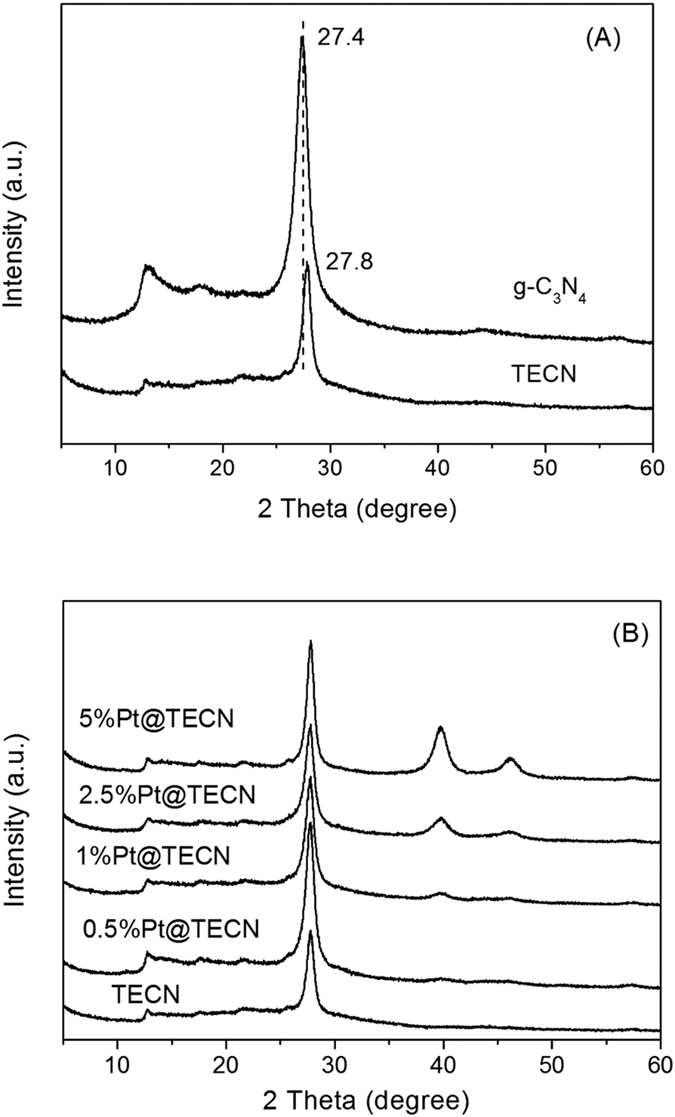
XRD patterns of (A) bulk g-C_3_N_4_ and TECN, (B) Pt@TECN catalysts with different of Pt loading.

**Figure 5:**
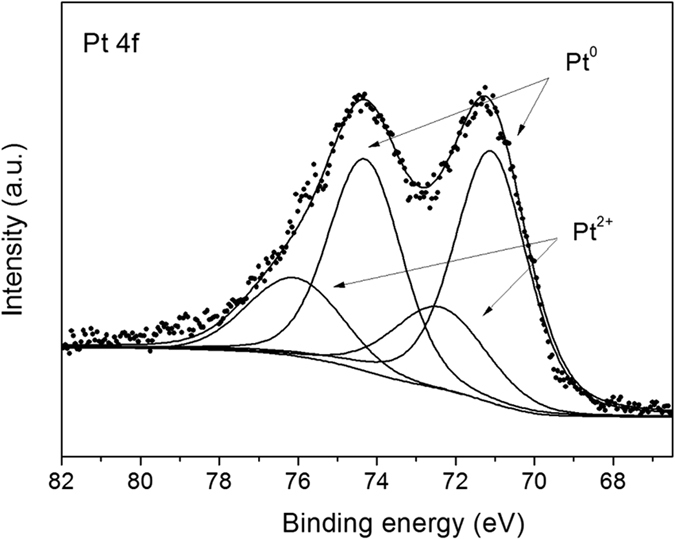
XPS spectra in the Pt 4f region of 5%Pt@TECN.

**Figure 6:**
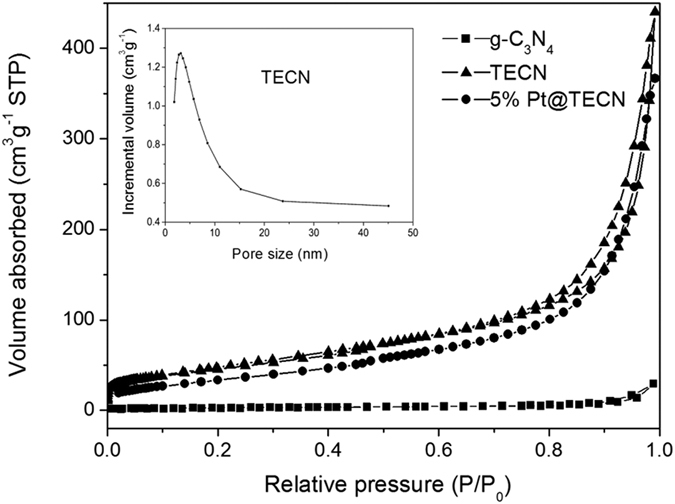
N_2_ adsorption–desorption isotherm g-C_3_N_4_, TECN and 5% Pt@TECN catalyst and the pore size distribution of TECN (inset).

**Figure 7:**
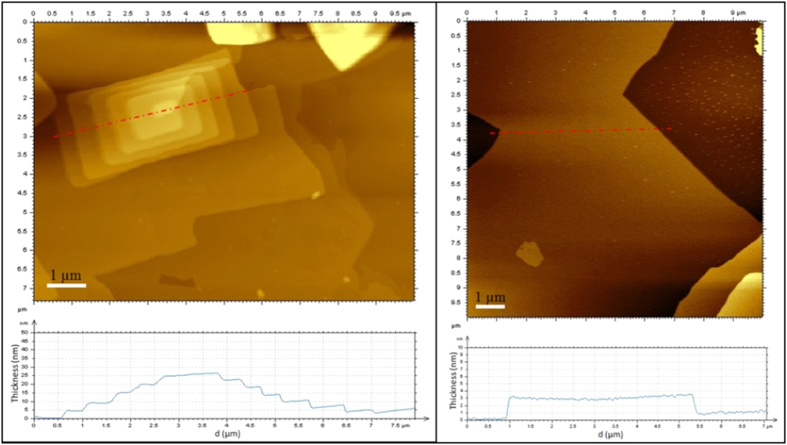
Tapping mode AFM images of bulk g-C_3_N_4_ (left) and TECN (right) samples and their corresponding thickness analyses.

**Figure 8:**
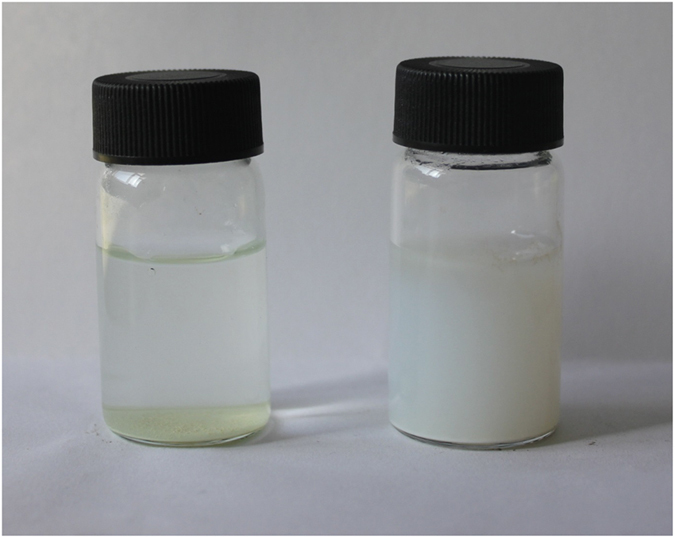
Photographs of the dispersions of bulk g-C_3_N_4_ (left) and TECN (right) in water, after sonication for 10 min and storage for 6 h under ambient conditions.

**Figure 9:**
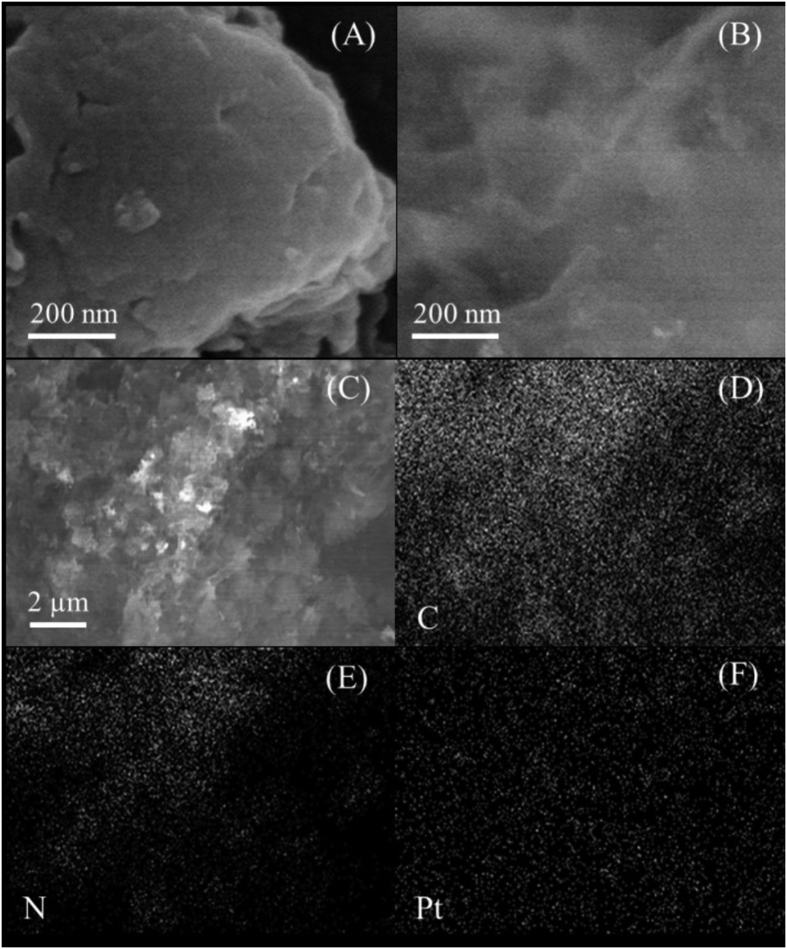
Typical SEM images of (A) bulk g-C_3_N_4_, (B,C) 5%Pt@TECN sample and the corresponding elemental mapping images of (D) carbon, (E) nitrogen and (F) platinum

**Figure 10:**
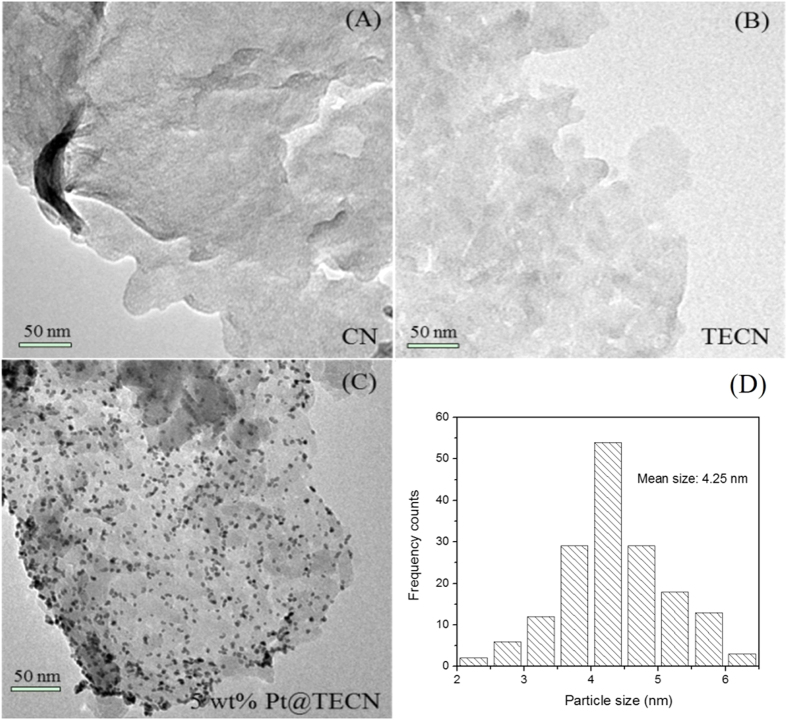
TEM images of (A) bulk g-C_3_N_4_, (B) TECN, (C) 5%Pt@TECN samples and (D) the size distribution of Pt particles (the total number of particles counted was 166)

**Figure 11:**
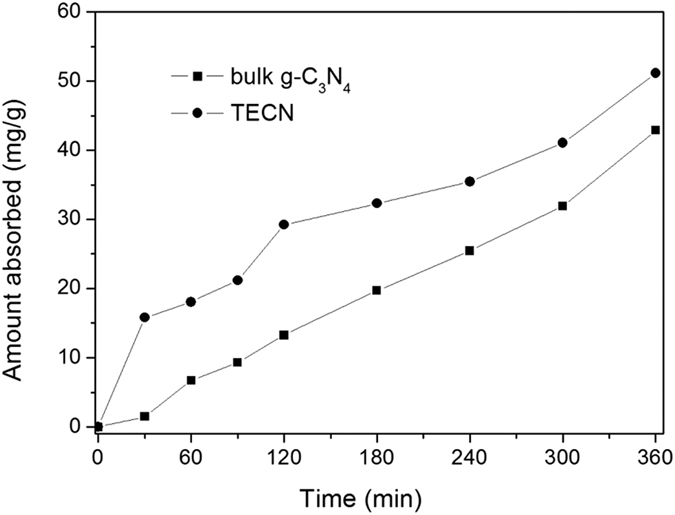
The amount of furfural absorbed on bulk g-C_3_N_4_ and TECN.

**Figure 12:**
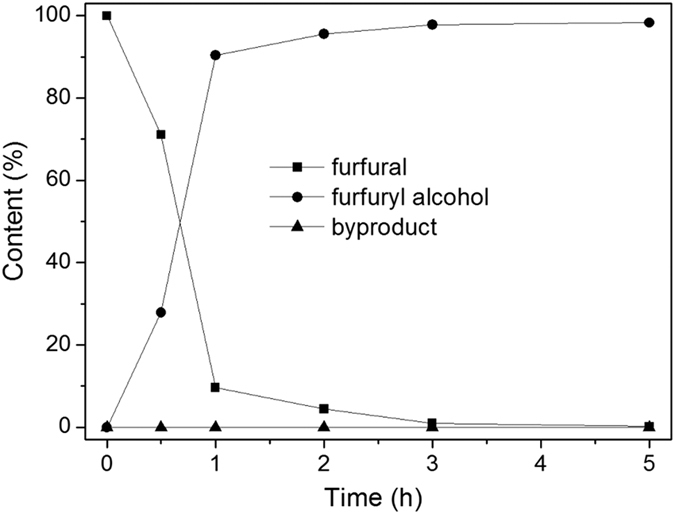
Evolution of reactant and product concentrations at 100 °C with reaction time over 5%Pt@TECN.

To illustrate the usefulness of the carbon nitride nanosheets supported Pt catalysts, the reduction of furfural with H_2_ was used as a model reaction. The reduction of furfural theoretically would probably proceed in four ways (see Fig. 1): i) the hydrogenation of C=O bond, ii) the hydrogenation of the furan ring, iii) the hydrogenolysis of the C=O bond, iv) decarbonylation. The reaction was firstly conducted at 100 °C under 1 MPa H_2_ in water. When 5%Pt@g-C_3_N_4_ was used as the catalyst, furfuryl alcohol was found to be the main product with 60.2% yield based on furfural within 5 h (Entry 3, Table 1). No other byproducts was observed, implying that the reaction might mainly proceed via chemoselective hydrogenation of C=O bonds to form furfuryl alcohol. This result suggested that g-C_3_N_4_ supported Pt material was a good hydrogenation catalyst, preferential to produce a furfuryl alcohol. Reference experiments showed that almost no reaction proceed with the system in absence of catalyst or in the presence of TECN support (Entry 1, 2, Table 1), indicating that Pt nanoparticles were the reactive metals in the hydrogenation reaction. As the surface area is a key factor for catalysis, when g-C_3_N_4_ nanosheets was used as the support for loading Pt nanoparticles, significant improvement of activity with complete conversion and high selectivity of furfuryl alcohol of >99% could be achieved in 5 h (Entry 7, Table 1). The NMR analysis also supported that the products of furfural hydrogenation in water was mainly furfuryl alcohol (see Figure S4). Figure 12 displayed the evolution of the furfural and product concentration with the time over 5%Pt@TECN in water. As shown in Fig. 12, in the initial stages of the reaction, furfural was transformed to furfuryl alcohol with a relative moderate rate, and about 29% conversion of furfural was obtained within 0.5 h. When the reaction time proceeded, the reaction was accelerated and almost 90% conversion of furfural was obtained within 1 h. The enhanced rate was presumably due to the activation of Pt@TECN by reduction of Pt^2+^ to Pt^0^ in the initial experiment. The result was partly supported by XPS analysis, which showed that the Pt^0^ content in the used catalyst was higher than that in the fresh catalyst (see Figure S2 in the supplementary information). As the reaction continues, the concentration of furfural decreased gradually, while the concentration of furfuryl alcohol increased correspondingly. After reaction for 5 h, furfural was converted completely to furfuryl alcohol with about 99% yield via hydrogenation. It was worth mentioning that no other product was observed during the whole process. The results demonstrated that furfural was mainly reacted with H_2_ by hydrogenation of C=O bond to form furfuryl alcohol in water and Pt@TECN was a good hydrogenation catalyst.

For comparison, Pt nanoparticles were also loaded in activated carbon and were used as the catalysts in furfural hydrogenation. As shown in Table S1, Pt@TECN exhibited higher activity than Pt@C (50.6% yield of furfural alcohol within 1 h) under identical conditions. The results implied that the nature of the support had a noticeable impact in the catalytic activity of Pt catalysts for furfural hydrogenation.

The impact of Pt content on the hydrogenation of furfural was investigated in the similar condition. As the Pt contents increased from 0.5 wt% to 5.0 wt%, the conversion of furfural after 5 h enhanced from 32.1% to 99% with high furfuryl alcohol selectivity of >99% (Entries 4–7, Table 1). Moreover, 0.5%Pt@TECN displayed more than three times higher TOF of 242 h^−1^ than that of 5%Pt@TECN (75 h^−1^). The effect of reaction temperature and H_2_ pressure was also studied. When 5%Pt@TECN was used as catalyst, with the elevated temperature from 80 °C to 100 °C, the furfural conversion after 1 h was enhanced evidently from 31.8% to 90.3% without any loss of selectivity (Entries 1–2, Table 2). Further increasing temperature to 120 °C did not enhance the activity obviously, and small amount of 2-methyl furan (~1.0%) was detected (Entry 3, Table 2). The H_2_ pressure also had a considerable effect on the reaction conversion. With the elevated H_2_ pressure from 0.5 MPa to 2.0 MPa, the furfural conversion was improved from 61.2% to 98% with high furfuryl alcohol selectivity (~99%, Entries 2, 4–5, Table 2). Small amount of 2-methyl furan (~1.1%) was also found under 2.0 MPa H_2_, implying that higher temperature and higher H_2_ pressure would result in hydrogenolysis of the C-O bond.

The choice of solvents usually has an impact on the catalytic activity in a liquid-phase reaction. In order to study the effect of solvents on the catalytic performance of 5%Pt@TECN, water, ethanol, toluene, isopropanol and octane were used as the solvents for furfural hydrogenation. The results were summarized in Table 3. Although the mechanism of the solvent effect on the heterogeneous catalysis was not clearly explicit, it has been considered that the activity was probably correlated with solvent polarity^34^. In our catalytic system, it was found that the catalytic activity was strongly influenced by the solvent. Among these solvents examined, the reaction in water showed the best activity for furfural hydrogenation, giving >99% yield toward furfuryl alcohol after 5 h (Entry 1, Table 3). The reaction in ethanol, toluene and isopropanol obtained relative temperate furfural conversion of 51.2–91.5% after 5 h, with the desired furfuryl alcohol selectivity of 70.5–96.7% (Entries 2–4, Table 3). The acetalization of furfural was also observed in ethanol with 29.5% selectivity of 2-furaldehyde diethyl acetal. When isopropanol was used as solvent, small amount of 2-isopropoxymethylfuran was formed with 3.3% selectivity. When toluene was used as solvent, furan was detected as a by-product with selectivity of 4.4%. Additionally, the reaction in octane exhibited poor activity with 8.3% conversion, 65.8% selectivity of furfuryl alcohol and 34.2% selectivity of furan (Entry 5, Table 3). These results indicated that Pt@TECN was not effective on hydrogenation reaction in the nonpolar solvent. Similar observations were made by Merlo *et al*. with Pt/SiO_2_ as the catalyst in non-polar solvents, which showed that non-polar toluene and hexane resulted in low furfural conversion and the formation of decarbonylation to furan^9^. It can be considered that hydrogenation of furfural to furfuryl alcohol with Pt@TECN catalyst was more easily to take place on polar solvent, particularly in strong polar solvent. Thus, the activity of Pt@TECN was closed related with the solvent, and water was the most suitable solvent for the hydrogenation of furfural in the system.

**Figure 13:**
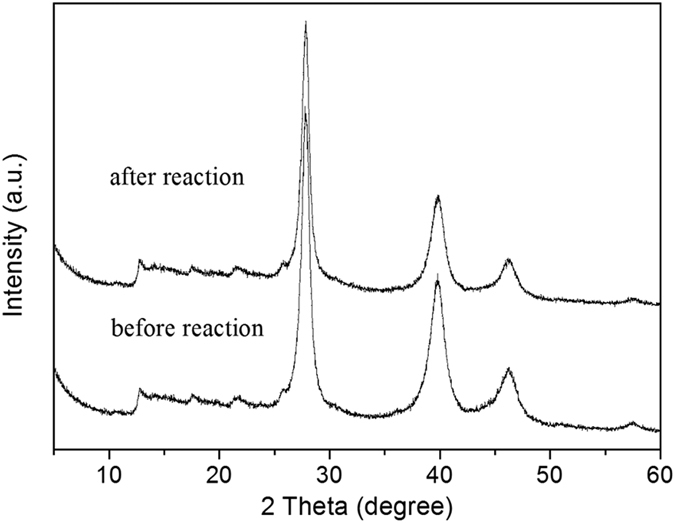
XRD patterns of 5%Pt@TECN catalyst before and after reaction.

It is important to investigate the reusability of the catalyst for the heterogeneous catalytic system, which is a key threshold feature for catalysts in industrial applications. On account of this viewpoint, the capability of recycling 5%Pt@TECN catalysts was studied by the hydrogenation of furfural at 100 °C in water. After reaction, the catalyst was recovered by filtration, followed by washing with water for several times and drying at 60 °C. As shown in Table 1, the 5%Pt@TECN could be reusable for four times without obvious loss of yield and selectivity for furfuryl alcohol (Entries 8–10, Table 1). ~98% of furfuryl alcohol yield with fresh 5%Pt@TECN remained in the fourth run. The slight decrease of the activity was probably due to the loss of catalyst mass during the recovery operations. Moreover, no dissolved Pt was detected in the reactant mixture after reaction, which was measured by AAS. The elemental analysis showed that the atomic ratios of C and N before and after reaction maintained 0.68. XRD analysis (Fig. 13) showed that the structure of carbon nitride nanosheets support and the size of Pt nanoparticles in the used catalyst was almost the same as those in the fresh one, reflecting the high durability of Pt@TECN catalyst. The used catalyst was also studied by XPS. As shown in Figure S2, there is no noticeable change in XPS spectra for the catalyst before and after the reaction, except the increase of Pt^0^ content. As calculated by XPS results, the atom ratios of Pt^0^ in the fresh 5%Pt@TECN increased from 71.3% to 77% after reaction. The increase of Pt^0^ content may be caused by partial reduction of Pt^2+^ during the reaction. These results illustrated that the carbon nitride nanosheets supported Pt nanoparticles were water tolerant, effective and stable catalysts for hydrogenation of furfural in water.

**Figure 14:**
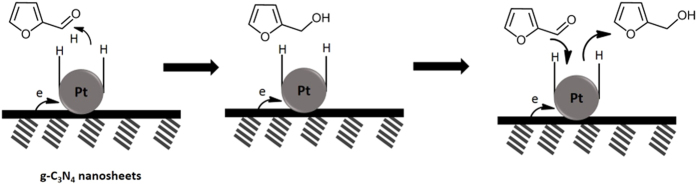
Possible reaction mechanism of the selective hydrogenation process over Pt@TECN.

Based on the above experimental results, a possible reaction pathway of the chemoselective hydrogenation process catalyzed by Pt@TECN catalyst in water was proposed in Fig. 14. The reduction of furfural to furfuryl alcohol would proceed in several consecutive steps. In the first stage, the furfural substrate was adsorbed on the surface of Pt@TECN catalyst and interacted with Pt surface via the C=O bond. Meanwhile, hydrogen molecule was adsorbed and decomposed into hydrogen atoms on electronically Pt surfaces. In the second, the C=O bond would be hydrogenated selectively to form C-OH by the attack of the activated hydrogen atoms. As 2D layered nanomaterials offered large specific surface area and small sheet thickness of about 3 nm, the diffusion of reactant and product could be accelerated in the nanosheets. Thus, once furfuryl alcohols were formed on the surface of catalyst, they would leave quickly and then be replaced by new reactant molecules. The good dispersibility of nanosheets in water favored for the hydrogenation reaction in water. Moreover, the 2D layered nanostructure would provide abundant reactive sites for adsorbing reactant molecules and enhance the adsorption ability of furfural substrates, which was demonstrated by furfural adsorption experiments. The nanostructure also facilitated the highly dispersion of Pt nanoparticles on its surface. These properties all co-contributed to the significant improvement of catalytic activity of the carbon nitride nanosheets supported Pt nanoparticles.

## Conclusions

In summary, the Pt@TECN catalysts showed superior activity in the chemoselective hydrogenation of furfural toward furfuryl alcohol in water. Meanwhile, they exhibited highly stability and reusability in the furfural hydrogenation in aqueous phase. The considerable catalytic performance originated from the structural features of carbon nitride and 2D layered nanomaterials. The nanosheets had a large surface area and small sheet thickness of ~3 nm, which made small-size Pt nanoparticles well distributed on the surface of the carbon nitride. The rich functional groups such as NH_2_, NH groups on the surface of carbon nitride nanosheets also led to a very stable and uniform dispersion of Pt nanoparticles. What is more, the nanosheets would provide abundant reactive sites for adsorbing reactant molecules and the rapid diffusion of reactant molecular and product.

## Methods

### Preparation of g-C_3_N_4_ nanosheets

Bulk g-C_3_N_4_ was synthesized by polycondensation of dicyandiamide under a semi-closed system. In a typical process, dicyandiamide (5.0 g) in a crucible was calcined at 550 °C for 5 h, and then cooled naturally to room temperature in static air. The resultant yellow powder of bulk g-C_3_N_4_ was formed. The g-C_3_N_4_ nanosheets were prepared from the as-prepared bulk g-C_3_N_4_ using by a thermal oxidation etching method. In detail, the bulk material (1 g) was put into a crucible without the cover and annealed at 500 °C for 2 h in static air. Light yellow g-C_3_N_4_ nanosheets were obtained with a yield of about 20%.

### Preparation of Pt@g-C_3_N_4_ nanosheets

Pt nanoparticles was deposited on TECN by an ultrasound-assisted reduction method. Typically, 0.1 g of as-prepared TECN was dispersed in 10 mL deionized water with sonication for 15 min. Then, H_2_PtCl_6_ (0.5 wt%, 0.5 mg Pt; 1 wt%, 1 mg Pt; 2.5 wt%, 2.5 mg Pt; 5 wt%, 5 mg Pt) aqueous solution was added into the resultant mixture. After sonication for another 15 min to reach the saturated adsorption of PtCl_6_^2−^ on the surface of carbon nitride, 16 mL NaBH_4_ solution (0.2% w/w) was added slowly to reduce Pt^2+^ to Pt^0^, followed by sonication for 30 min. Finally, the product was separated by filtration, washed with water and dried at 40 °C. For comparison, bulk g-C_3_N_4_ supported Pt catalyst and activated carbon supported Pt catalyst were prepared in a similar procedure and denoted as Pt@gCN and Pt@C, respectively.

### Characterizations

The X-ray diffraction (XRD) patterns of all samples were measured by using a Bruker D8 Advance X-ray diffraction diffractometer with CuKa radiation (λ = 1.5147 Å). The morphology of g-C_3_N_4_ nanosheets and supported Pt samples were determined by using a H-7600 transmission electron microscopy (TEM) and a field emission Hitachi S-4800 scanning electron microscope (SEM). The atomic force microscopic (AFM) images of carbon nitride were measured by a MultiMode Nanoscope V scanning probe microscopy system (Agilent 5400) in the tapping mode. The textural properties of samples were studied by N_2_ adsorption/desorption isotherm measurements at 77 K using a micromeritics ASAP 2020 m+c sorptometer. The samples were degassed in vacuum at 150 °C for 6 h prior to measurement. The Brunauer-Emmet-Teller (BET) method was used to determine the specific surface area. X-ray photoelectron spectroscopy (XPS) data was collected on a Thermo ESCALAB250 instrument with a monochromatized Al Ka line source. All the binding energies were calibrated according to C 1s peak at 284.6 eV. Elemental analysis was conducted on Vario El elemental analyzer to determine the elemental composition of the samples. The Pt content in the Pt@TECN was determined by an INESA 4510F atomic absorption spectrometer (AAS).

### Furfuryl alcohol adsorption

150 mg of carbon nitride material was added into a 50 mL of furfuryl alcohol aqueous solution with an initial concentration of 500 ppm and stirred at room temperature. At a given time, 1.5 mL of the solution was removed and centrifuged. The supernatant was then added with methanol by the volume of 1:1 and then analysed by a gas chromatograph (VARIAN 450). The amount of furfuryl alcohol adsorption was calculated according to the formula: the amount of furfuryl alcohol adsorption 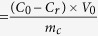. Here C_0_ is the initial concentration of furfuryl alcohol, C_r_ is the residual furfuryl alcohol concentration after adsorption, V_o_ is the volume of aqueous solution, m_c_ is the mass of sample.

### Catalytic activity for furfural hydrogenation

The reduction of furfural with H_2_ was chosen as a model reaction to evaluate the activity of the Pt catalysts. The reaction was conducted in a sealed stainless steel reactor (50 mL). After sealing the reactor was firstly purged with 2 MPa H_2_ several times to remove air, and H_2_ was pressurized to the desired pressure. Then the reactor was heated to the desired temperature and stirred at 600 rpm. In a typical procedure, Pt catalyst (0.24 mol% Pt relative to substrate), 20 mL deionized water and 0.4 mL furfural were mixed and added into the reactor. The mixture was heated to 100 °C under 1 MPa H_2_ and stirred for desired time. After the reaction, the reaction mixture was cooled to room temperature and depressurized. The reaction mixture was extracted with ethyl acetate. Then 5 times of ethanol was added to the aqueous phase in order to form one solution. The products in both the organic and aqueous phases were analyzed with n-butyl alcohol as the internal standard by a gas chromatograph (VARIAN 450). Pure chemicals, a GC-MS and NMR (Bruker AVANCE III 600) analysis were used to identify the products (see supplementary information). The conversions were calculated on the amount of reactant added into the reactor. The mass balance of furfural and detected products was >98%.

## Additional Information

**How to cite this article**: Chen, X. *et al*. Highly selective hydrogenation of furfural to furfuryl alcohol over Pt nanoparticles supported on g-C_3_N_4_ nanosheets catalysts in water. *Sci. Rep.* **6**, 28558; doi: 10.1038/srep28558 (2016).

## Supplementary Material

Supplementary Information

## Figures and Tables

**Table 1:** Furfural hydrogenation over Pt catalysts after 5 h reaction in water at 100 °C^a^.

Entry	Catalyst	Conv. (%)	Sel. (%)	TOF^b^ (h^−1^)
1	no catalysts	<1	–	–
2	TECN	<1	–	–
3	5% Pt@CN	60.9	>99	46
4	0.5% Pt@TECN	32.1	>99	242
5	1% Pt@TECN	49.4	>99	186
6	2.5% Pt@TECN	95.9	>99	145
7	5% Pt@TECN	>99	>99	75
8	Reuse entry 7 for 2^nd^	>99	>99	75
9	Reuse entry 7 for 3^rd^	98.5	>99	75
10	Reuse entry 7 for 4^th^	97.8	>99	74

^a^Reaction conditions: 50 mg catalyst, 20 mL H_2_O, 0.4 mL furfural, 1.0 MPa H_2_, 100 °C, 5 h.

^b^TOF = [reacted mol furfural]/[(total mol metal) * (reaction time)].

**Table 2:** Furfural hydrogenation over 5% Pt@TECN catalyst after 1 h reaction in water^a^.

Entry	Catalyst	T(°C)	Time (h)	Conv. (%)	furfuryl alcohol sel. (%)	2-methyl furan Sel. (%)
1	5% Pt@TECN	80	1	31.8	>99	0
2	5% Pt@TECN	100	1	90.3	>99	0
3	5% Pt@TECN	120	1	90.5	99	1.0
4	5% Pt@TECN^b^	100	1	61.2	>99	0
5	5% Pt@TECN^c^	100	1	98.0	98.9	1.1

^a^Reaction conditions: Pt@TECN (0.24 mol% Pt relative to substrate), 20 mL H_2_O, 0.4 mL furfural, 1.0 MPa H_2_.

^b^0.5 MPa H_2_.

^c^2.0 MPa H_2_.

**Table 3:** Effect of solvent on furfural hydrogenation over 5%Pt@TECN after 5 h reaction at 100 °C^a^.

Entry	Solvent	Conv. (%)	Furfuryl alcohol Sel. (%)	Furan Sel. (%)	SP^b^ Sel. (%)	Solvent polarity
1	Water	>99	>99	0	0	10.2
2	Ethanol	67.4	70.5	0	29.5	4.3
3	Toluene	51.2	95.6	4.4	0	2.4
4	Isopropanol	91.5	96.7	0	3.3	3.9
5	Octane	8.3	65.8	34.2	0	0

^a^Reaction conditions: Pt@TECN (0.24 mol% Pt relative to substrate), 20 mL solvents, 0.4 mL furfural, 1.0 MPa H_2_, 100 °C, 5 h.

^b^2-furaldehyde diethyl acetal (ethanol) and 2-isopropoxymethylfuran (isopropanol) expressed as Solvent Product (SP).
