# Supplementary material for: Highly selective hydrogenation of furfural to furfuryl alcohol over Pt nanoparticles supported on g-C3N4 nanosheets catalysts in water
Source: Sci Rep. 2016 Jun 22;6:28558. doi: 10.1038/srep28558 (PMC4916514; doi:10.1038/srep28558)
Supplement: Supplementary Information [file srep28558-s1.pdf]

Electronic Supplementary Information

Highly selective hydrogenation of furfural to furfuryl alcohol over Pt nanoparticles supported on g-C_3N_4 nanosheets catalysts in water

Xiufang Chen*, Ligang Zhang, Bo Zhang, Xingcui Guo, Xindong Mu*

Key Laboratory of Bio-based Materials, Qingdao Institute of Bioenergy and Bioprocess Technology, Chinese Academy of Sciences, Qingdao 266101, China

[*] Correspondence and requests for materials should be addressed to X.C. (email: chenxf@qibebt.ac.cn) or X.M. (email: muxd@qibebt.ac.cn)

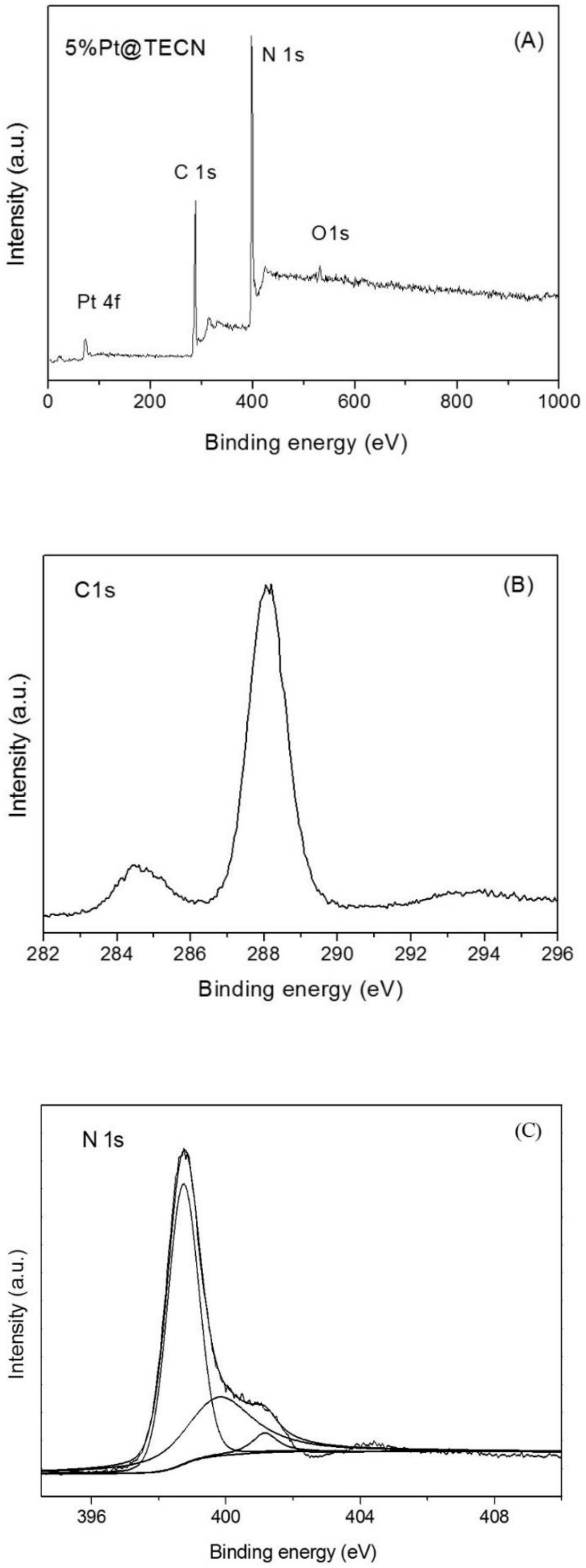

Figure S1 (A) XPS survey spectrum of 5%Pt@TECN catalyst, XPS spectra of (B) C1s, (C) N 1s.

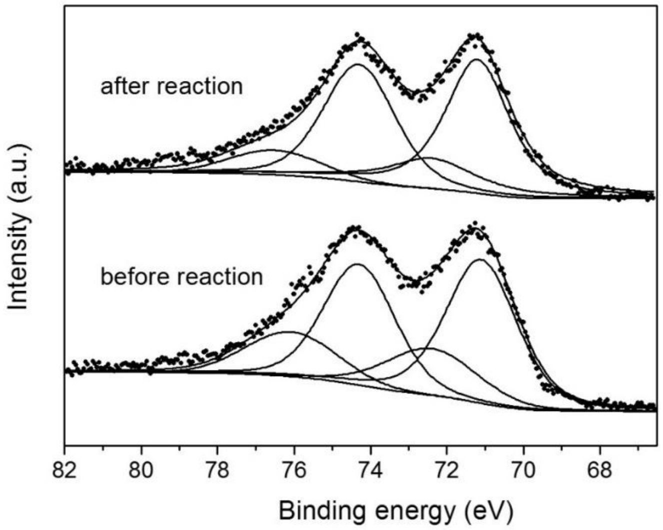

Figure S2. XPS spectra in the Pt 4f region of fresh and spent 5% Pt@TECN catalysts.

Table S1. Furfural hydrogenation over Pt catalysts after 1 h reaction in aqueous phase at 100 ^{o}C.

Entry	Catalyst	Conv. (%)	Furfuryl alcohol Sel. (%)
1	5% Pt@TECN	90.3	>99
2	5% Pt@C	51.1	>99

Reaction conditions: Pt catalyst (0.24 mol% Pt relative to substrate), 20 mL H_2O, 0.4 mL furfural, 1.0 MPa H_2, 100 ^{o}C, 1 h.

Gas-chromatographic analysis of the reaction mixture form Table 1, entry 7 and Table 2, entry 1.

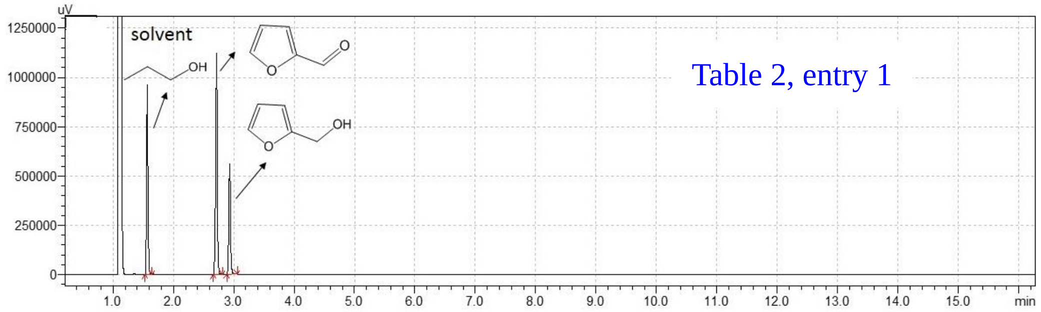

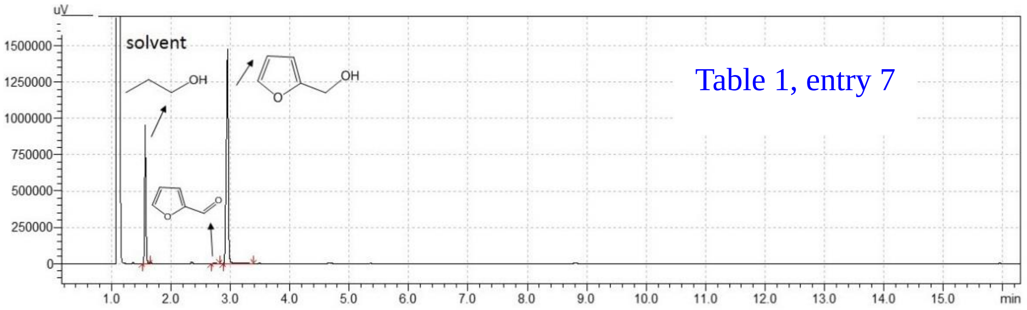

Figure S3. Representative GC spectrums of furfural hydrogenation in water.

NMR analysis of the reaction mixture from Table 1, entry 7.

The reaction mixture was obtained by furfural hydrogenation over 5%Pt@TECN under 1MPa H_2 for 5 h in water (Table 1, entry 7). Then the reaction mixture was extracted with EtOAc, the organics were dried (Na_2SO_4), concentrated and analysed by NMR. The NMR analysis (see figure S4) confirmed that the main product of the reaction was furfuryl alcohol.

^{1}H NMR (600 MHz, $CDCl_3$) δ 7.36 (d, 1H), 6.30 (t, 1.08H), 6.24 (d, 1.06H), 4.51 (d, 2.1H), 3.22 (br s, 0.98H).

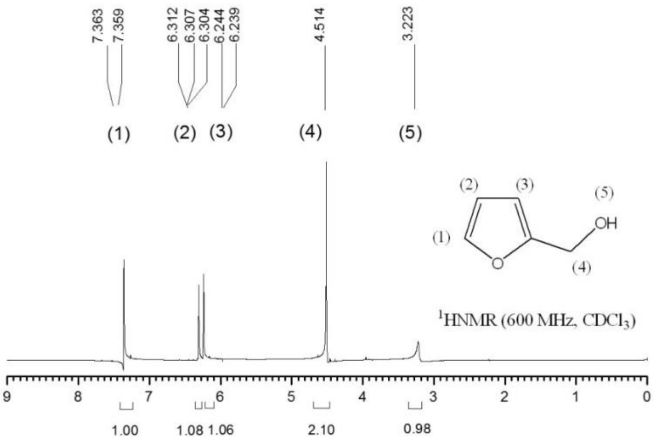

Figure S4. The ^{1}H NMR spectra of the reaction mixture.

Quantitative GC analyses

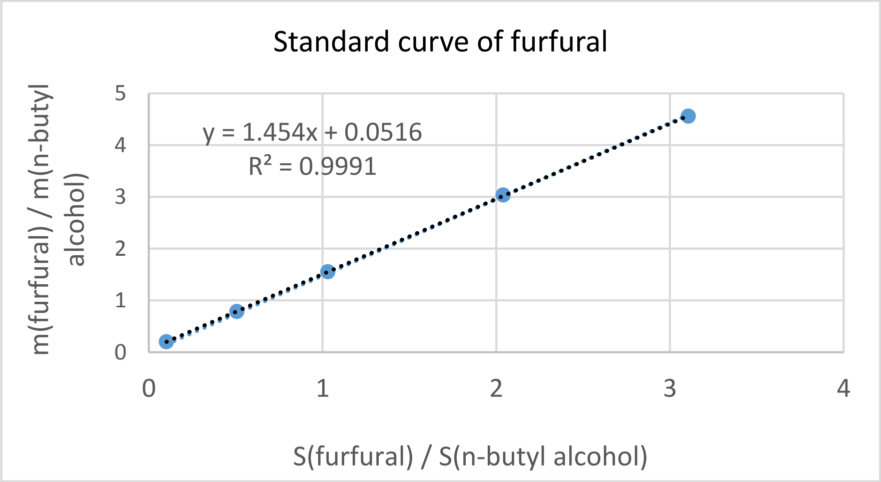

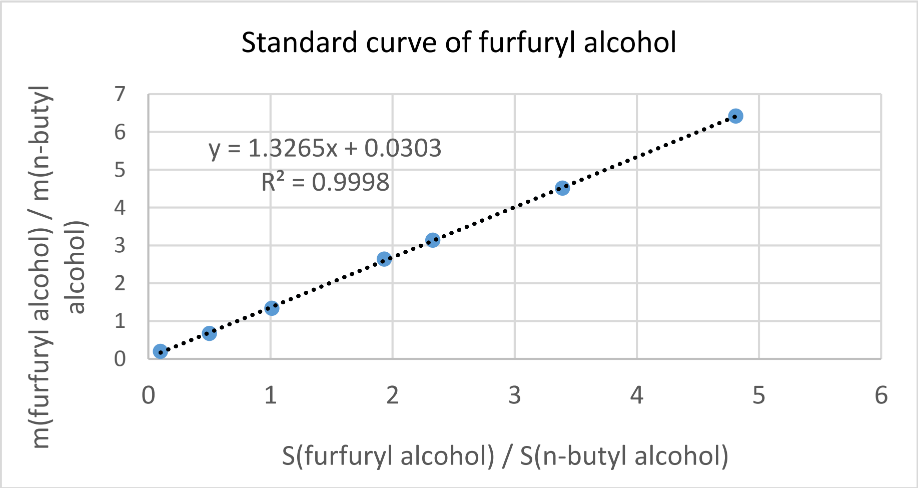

Figure S5. Standard curve of furfural with n-butyl alcohol and furfuryl alcohol with n-butyl alcohol.
